# Remodeling of the gut microbiome during Ramadan-associated intermittent fasting

**DOI:** 10.1093/ajcn/nqaa388

**Published:** 2021-04-12

**Authors:** Junhong Su, Yueying Wang, Xiaofang Zhang, Mingfu Ma, Zhenrong Xie, Qiuwei Pan, Zhongren Ma, Maikel P Peppelenbosch

**Affiliations:** Department of Gastroenterology and Hepatology, Erasmus MC—University Medical Center Rotterdam, Rotterdam, The Netherlands; Department of Basic Medicine, Medical School, Kunming University of Science and Technology, Kunming, China; China-Malaysia National Joint Laboratory, Biomedical Research Center, Northwest Minzu University, Lanzhou, China; Department of Basic Medicine, Medical School, Kunming University of Science and Technology, Kunming, China; The Fifth People's Hospital of Qinghai (Qinghai Tumor Hospital), Xining, China; The Medical Biobank, the First Affiliated Hospital of Kunming Medical University, Kunming, China; Department of Gastroenterology and Hepatology, Erasmus MC—University Medical Center Rotterdam, Rotterdam, The Netherlands; China-Malaysia National Joint Laboratory, Biomedical Research Center, Northwest Minzu University, Lanzhou, China; Department of Gastroenterology and Hepatology, Erasmus MC—University Medical Center Rotterdam, Rotterdam, The Netherlands

**Keywords:** intermittent fasting, healthy volunteers, gut microbiota, Lachnospiraceae, butyrate, calorie intake, Ramadan

## Abstract

**Background:**

Intermittent fasting is a popular dietary intervention with perceived relatively easy compliance and is linked to various health benefits, including weight loss and improvement in blood glucose concentrations. The mechanistic explanations underlying the beneficial effects of intermittent fasting remain largely obscure but may involve alterations in the gut microbiota.

**Objectives:**

We sought to establish the effects of 1 mo of intermittent fasting on the gut microbiome.

**Methods:**

We took advantage of intermittent fasting being voluntarily observed during the Islamic faith-associated Ramadan and sampled feces and blood, as well as collected longitudinal physiologic data in 2 cohorts, sampled in 2 different years. The fecal microbiome was determined by 16S sequencing. Results were contrasted to age- and body weight–matched controls and correlated to physiologic parameters (e.g., body mass and calorie intake).

**Results:**

We observed that Ramadan-associated intermittent fasting increased microbiome diversity and was specifically associated with upregulation of the Clostridiales order–derived Lachnospiraceae [no fasting 24.6 ± 13.67 compared with fasting 39.7 ± 15.9 in relative abundance (%); linear discriminant analysis = 4.9, *P* < 0.001 by linear discriminant analysis coupled with effect size measurements] and Ruminococcaceae [no fasting 13.4 ± 6.9 compared with fasting 23.2 ± 12.9 in relative abundance (%); linear discriminant analysis = 4.7, *P* < 0.001 by linear discriminant analysis coupled with effect size measurements] bacterial families. Microbiome composition returned to baseline upon cessation of intermittent feeding. Furthermore, changes in Lachnospiraceae concentrations mirrored intermittent fasting–provoked changes in physiologic parameters.

**Conclusions:**

Intermittent fasting provokes substantial remodeling of the gut microbiome. The intermittent fasting–provoked upregulation of butyric acid–producing Lachnospiraceae provides an obvious possible mechanistic explanation for health effects associated with intermittent fasting.

## Introduction

See corresponding article on page 1075.

Intermittent fasting, a dietary concept in which individuals repeatedly, voluntarily, and severely restrict food intake for approximately 16 to 24 h, is widely practiced for a variety of religious, social, and medical reasons (1). Many health benefits are attributed to practicing intermittent fasting; for example, the American Heart Association claims that intermittent fasting may produce weight loss, reduce insulin resistance, and lower the risk for cardiometabolic diseases (2). The mechanisms mediating the effects of intermittent fasting remain, however, largely obscure, hampering the development of rational strategies to use intermittent fasting for improved health and treatment of disease.

Experiments in experimental rodents and observations in human volunteers or patients suggest that the beneficial effects of intermittent fasting can only partly be explained by reduced calorie intake (3). A plethora of alternative mechanisms mediating the effects of intermittent fasting have been brought forward and can roughly be grouped in 3 categories involving mechanisms involving circadian biology, altered lifestyle, and remodeling of the gut microbiome (4). The notion that the latter is especially instrumental for mediating the beneficial effects of intermittent fasting is supported by many observations in experimental animals, including that intermittent fasting–provoked white adipose tissue browning (5) requires an intestinal flora (6) or that restructuring of the gut microbiome by intermittent fasting counteracts retinopathy in diabetic mice (7). Also, in a murine model of multiple sclerosis, intermittent fasting–evoked reduced autoimmunity is critically dependent on microbiome alterations (8). The effects of intermittent fasting on the human microbiome remain, however, largely uncharacterized, and in view of the problems associated with extrapolating data in experimental rodents to humans (9), it would be important to establish the effects of intermittent fasting in our species as well.

Prompted by the abovementioned considerations, we decided to characterize the effects of a monthly episode of intermittent fasting on the human gut microbiome and to contrast the results with nonfasting controls and with the effects of cessation of intermittent fasting following the intervention.

## Methods

### Young male adult cohort

We prospectively recruited male volunteers who expressed the intention to observe fasting in the month of Ramadan, according to the Islamic law. The entire fasting period lasted for 30 d and was performed during Ramadan, and the intermittent fasting period of each day was from dawn to sunset (which was approximately 16 h in this study). Volunteers were recruited from 2 independent locations (Najiaying town in Yuxi city and the Chenggong district in Kunming city), but both are located in Yunnan province, China. Through reviewing the medical history, only healthy individuals were selected. Exclusion criteria were *1*) obese [BMI (in kg/m^2^) ≥30] and *2*) self-reported diabetes, hypertension, arthritis, chronic respiratory disorders, or any gastrointestinal disorders. Prescription of antibiotics in the month prior to the start of the study was used as another exclusion criterion. In total, 30 volunteers were selected. The stool of each participant was collected on the first day of the 2016 Ramadan, at an intermediate time point (day 15), and on the final day (day 30) of this religious fasting period. To avoid environmental contamination, all volunteers were kindly requested to *1*) place a clean newspaper to catch the stool, *2*) wash their hands before sampling their own stool, and *3*) collect the stool in a clean, screw-top container by using the spoon that came with the container and then close the container by screwing the lid shut. Following collection, stool samples were transported on ice (using a polystyrene icebox) to the laboratory and immediately stored at –80°C until further processing. All samples spent no longer than 2 h on ice. All participants provided informed consent, and the study was approved by the relevant ethical committee.

### Middle-aged cohort

Participants were recruited from Xining city, which is located in Qinghai province in China. Volunteers who, upon review of their medical history, showed earlier diagnoses of metabolic disorder, cardiovascular disease, or chronic inflammatory disorders were excluded, as were those with a current acute inflammatory disorder. In addition, volunteers employing antibiotic use in the month prior to the study were excluded as well. Following review of the medical history, 37 healthy participants were selected and enrolled in this cohort. Twenty-seven of the selected participants subjected themselves to intermittent fasting, and the remaining 10 served as nonfasting controls. The stool samples for each participant were collected at T1 (start of the 2018 Ramadan), T2 (the end of the 2018 Ramadan), and T3 (1 mo following the end of Ramadan fasting). Environmental contamination for stool sampling was controlled by the same measures as described for the young population above. Following production, samples were transported in a Styrofoam icebox to the laboratory and immediately stored at –80°C. All samples spent no longer than 3 h on ice. During the study, 25 volunteers in the intermittent fasting group fully complied with the investigation protocol, but 2 volunteers failed to provide their fecal samples at T3. All volunteers in the nonfasting control group fully complied with the study protocol. Of note, all volunteers, including those in the unfasted group, were asked to provide their fecal and blood samples at each time point: before, the end, and 1 mo after the cessation of the 2018 Ramadan fasting. All participants provided informed consent, and the study was approved by the relevant ethical committee.

In this cohort, we also measured selected physiologic parameters. Changes in fat mass and fat percentage in these middle-aged participants during fasting and body composition indices were measured by a body composition analyzer (SH-900G; Novogene) at all 3 time points. For blood parameter analysis, following overnight fasting, blood was collected and subsequently sent to Zhejiang DIAN Diagnostics, and the following blood parameters were determined: aspartate aminotransferase, alanine aminotransferase, direct bilirubin, indirect bilirubin, total bilirubin, γ-glutamyltransferase, glucose, hemoglobin A1c, triglycerides, HDL cholesterol, LDL cholesterol, total cholesterol, creatinine, urea, and uric acid.

In addition, information on food intake was collected. To this end, we used a modified FFQ to assess dietary intake 1 mo before fasting, during fasting, and a month after the cessation of the fasting. There were 5 frequency categories in FFQ ranging from “never eaten” to “eating daily,” “eating one time per week,” “eating one time per month,” and “eating one time per year.” Participants reported their food intake frequency through a recall interview after intermittent fasting. In this study, we did not include any food types that were only consumed on a yearly basis. For dietary components with a self-reported consumption frequency of once per week or more, calorie intake was calculated according to the corresponding frequency. Finally, derived weekly energy and nutrient intake was calculated in accordance with the China Food Composition Database.

### Ethics

The protocol of this study was approved by the ethical committee of the medical faculty at Kunming University of Science and Technology (protocol 2017JC002; KMUST-MEC-017) and the medical ethical committee of Northwest Minzu University (XBMZ-YX-2,016,001). This study is registered at www.chictr.org.cn (ChiCTR2000034646). All volunteers provided written informed consent prior to the study.

### Outcome

The primary outcome was the change in gut microbiota composition after a 30-d Ramadan-associated intermittent fasting compared with baseline. To this end, gut microbiota were analyzed for diversity, composition, and taxonomic abundance. Secondary outcome variables were changes in body composition, blood parameters, and food intake.

### Next-generation sequencing and data processing

Fecal DNA was extracted from the stool sample using the QIAamp DNA Stool Mini Kit (Qiagen), according to previously described procedures (10). To minimize the impact of reagent microbiome (kitome), fecal DNA was prepared using the same batch of kits and reagents for each cohort. In addition, a blank sterile microcentrifuge tube was used as a negative control to characterize kitome influences during DNA extraction. DNA quality was monitored on 1% agarose gels (**Supplementary Figure 1**). As a control for bacterial environmental contamination during DNA library preparation, DNA-free water (blank library) was used. The quality of the library was assessed using the Qubit@ 2.0 Fluorometer (Thermo Scientific). Next-generation sequencing of the V3–4 region of the bacterial 16S ribosomal RNA gene was performed by the NovoGene Company. Data processing and analysis were performed according to routine procedures, which have been described elsewhere in detail (11–14).

### Statistical analysis

Gut microbiota diversity was estimated by calculating the Shannon and Simpson indices using alpha-diversity.py in QIIME (15), and the Wilcoxon signed-rank test was used to determine statistical significance between groups. Principal coordinates analysis (PCA) of Bray–Curtis distance was performed using the “vegan” package in R programming language (version 4.0.2) for establishing the shift of the gut microbiota composition after Ramadan-associated intermittent fasting. Multivariate data analysis according to analysis of similarities (ANOSIM) was applied to determine gut microbial shifts. A *P* value less than 0.05 was considered statistically significant. To identify bacterial taxa whose sequences were differentially abundant between groups, linear discriminant analysis (LDA) coupled with effect size measurements (LEfSe) analysis was applied with the significance level of 0.05 and the logarithmic LDA score threshold equal to 4 (http://huttenhower.sph.harvard.edu/galaxy). Phylogenetic Investigation of Communities by Reconstruction of Unobserved States (PICRUSt) was performed to identify functional genes in the sampled microbial community based on the Kyoto Encyclopedia of Genes and Genomes pathway database (16). Predicted metagenomics pathways were tested for potential significance by Student *t* test using the function t.test() in R. Differences in operational taxonomic unit (OTU) number and taxonomic relative abundance were analyzed by the Wilcoxon signed-rank test using the function wilcox.test() in R. Spearman correlation between gut microbiota and host markers was calculated using the function cor.test() in R. A *P* value of less than 0.05 was considered statistically significant.

### Availability of data and materials

The data sets used and/or analyzed during the current study are available from the corresponding author on reasonable request.

## Results

### Participants, sample collection, and study execution

**FIGURE 1:**
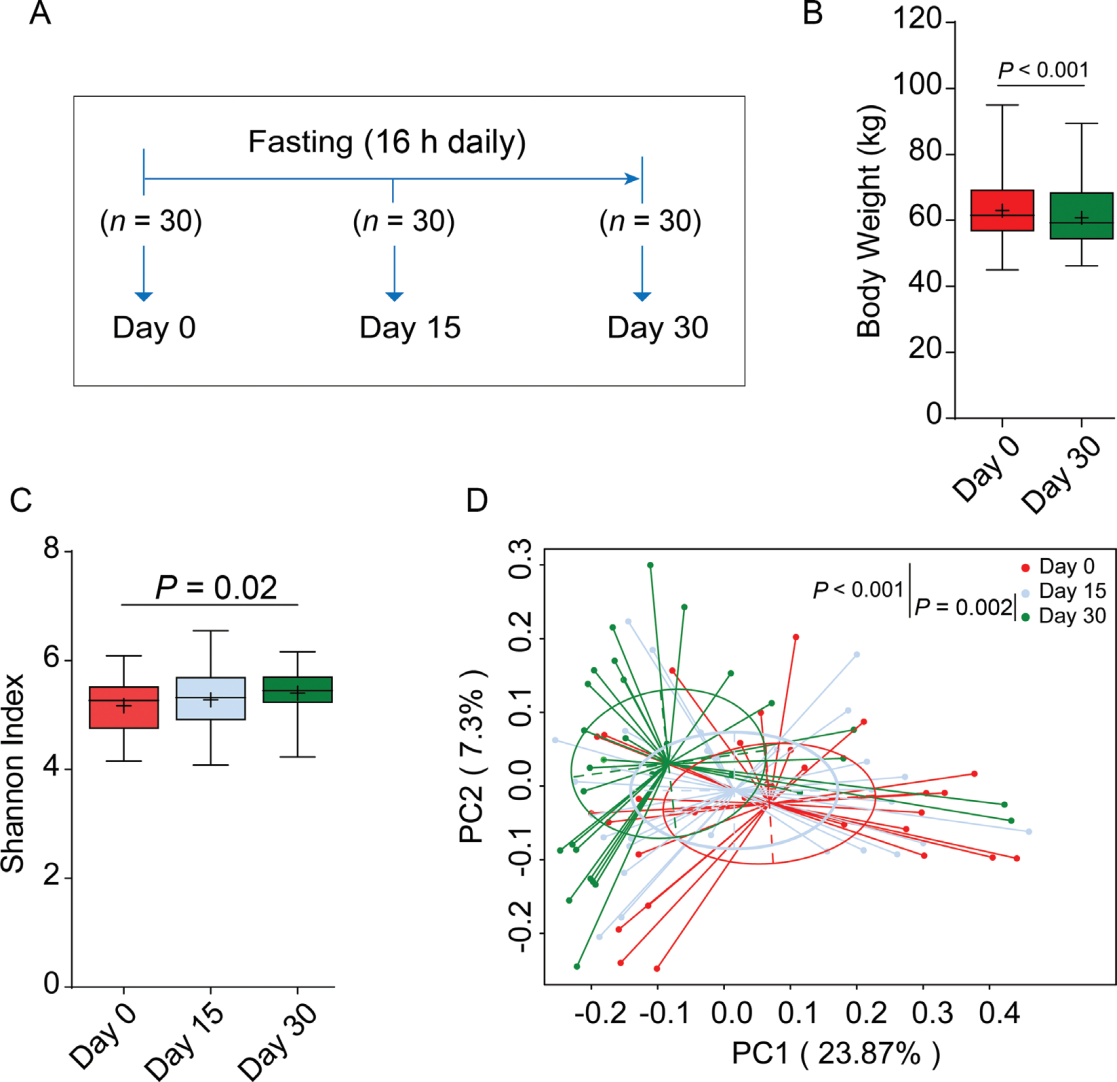
Intermittent fasting–shaped gut microbiota in a young adult cohort. (A) Study design of the cohort (*n* = 30). During the study, any individual who used antibiotics during fasting or missed 1 or more time points for fecal collection was excluded. Boxplot for body weight (B) and gut microbiota diversity (C) shows the minimum, the first quantile, median, mean (+), the third quantile, and the maximum values for samples at day 0, day 15, or day 30; *n* = 30 per time point. The diversity of gut microbiota was assessed by calculating the Shannon index. The significance between groups was estimated by using a 2-tailed paired Student *t* test. (D) A principal coordinates analysis was generated based on the Bray–Curtis distance. Each point corresponds to a community from a single individual. Colors indicate community identity. Statistically significant differences were calculated using an analysis of similarities test; *n* = 30 per time point.

**FIGURE 2:**
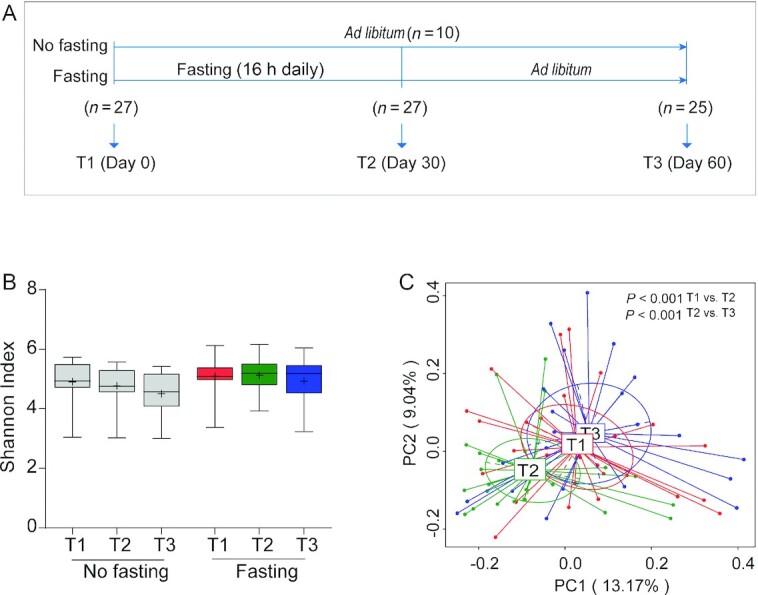
Intermittent fasting–shaped gut microbiota in a middle-aged cohort. (A) Study design of the cohort (no fasting, *n* = 10; fasting, *n* = 27). Fecal and blood samples were collected before (T1), after (T2), and 1 mo after the cessation of fasting (T3). At T3, 2 participants in the fasting group were not included for analysis due to unwillingness to continue the study. Another 2 participants failed to provide their fecal samples at this time point. (B) Boxplot for gut microbiota diversity, as determined by calculating the Shannon–Weaver index, shows the minimum, the first quantile, median, mean (+), the third quantile, and the maximum Shannon index values for samples at T1 (*n* = 10 for no fasting compared with *n* = 27 for fasting), T2 (*n* = 10 for no fasting compared with *n* = 27 for fasting), and T3 (*n* = 10 for no fasting compared with *n* = 23 for fasting). Significance between groups was estimated by using a 2-tailed paired Student *t* test for intragroup or Mann–Whitney test for intergroup testing. (C) Principal coordinates analyses on Bray–Curtis dissimilarities of bacterial communities from fasted participants at 3 time points are shown (*n* = 27 for T1 and T2; *n* = 23 for T3). Each point corresponds to a community from a single individual. Colors indicate community identity. Ellipses show the 95% confidence intervals. Differences in community shifts using an analysis of similarities test were indicated as well.

Ramadan-associated intermittent fasting is from dawn to sunset (∼16 h in the present study) and is typically adhered to for a consecutive 30 d. In this study, participants were kindly requested to restrain from overeating or binge eating at each meal during the Ramadan month. We first prospectively enrolled 30 healthy nonobese young men (average age 19 y) who expressed the intention to fast for 30 d during Ramadan. These volunteers were from Kunming city of Yunnan province in China (**Table 1**; **Supplementary Figure 2**), where white rice or rice noodles are staple foods and the mainstay of calorie intake. During the study, stool samples from each participant were collected at 3 time points to allow assessment of individual-specific dynamic changes in gut microbiome (**Figure 1**A). In addition, changes in selected metabolic parameters were assessed. We observed that 30 d of intermittent fasting caused a statistically significant loss of 3.45% in body weight in this young adult cohort (Figure 1B), which is comparable to what was observed by Stekovic et al. (17) after 4 wk of strict alternate-day fasting in a volunteer cohort. In addition, we included a middle-aged (on average 40 y old; **Table 2**) cohort from Xining city of Qinghai province in China who adhered to 30 d of Ramadan fasting in 2018 and compared the results with a nonfasting matched control group (participants matched by age, body weight, and BMI), which was sampled at the same time points as the fasting group (Table 2; **Supplementary Figure 3**). In this area, wheat flour is the most important staple food and calorie source. Fecal and blood samples were collected before (T1), after (T2), and 1 mo after the cessation of fasting (T3) (**Figure 2**A). Also, in this cohort, the Ramadan-inspired intermittent fasting provoked statistically significant weight loss (**Supplementary Figure 4A**), which was driven by a reduction in body fat (**Table 3**). It should be noted that the body fat mass of 3 of the fasting volunteers was much higher than that of the nonfasting volunteers at T1, thus leading to a higher average level of body fat in the Ramadan group. This effect was not statistically significant (Supplementary Figure 4B). In this cohort, we also analyzed blood enzymes and observed that especially liver enzymes improved during intermittent fasting, especially compared with nonfasting controls, in line with the beneficial effects associated with intermittent fasting in general (Table 3) (18). We thus concluded that our study cohorts would allow us to make meaningful statements on intermittent fasting–provoked changes in the microbiome and relate effects to changes in physiologic parameters.

**TABLE 1:** Characteristics of the young cohort^1^

Volunteer characteristics	Fasting
No.	30
Sex, % male	100
Age, y	18.63 ± 1.75

1 Data presented as counts (%) or mean ± SD.

**TABLE 2:** Characteristics of the middle-aged cohort^1^

Volunteer characteristics	No fasting	Fasting
No.	10	27
Sex, % male	70	37
Age, y	42.6 ± 7.9	39.9 ± 6.4
Body weight, kg	64.2 ± 10.2	64.1 ± 9.9
BMI, kg/m^2^	24.1 ± 2.3	24.3 ± 2.4

1 Data are presented as mean ± SD as appropriate.

**TABLE 3:** Body composition indices and blood parameters in the middle-aged cohort at T1, T2, and T3^1^

	No fasting			*P* value^2^			Fasting			*P* value^2^			*P* value^3^		
Variable	T1 (*n* = 10)	T2 (*n* = 10)	T3 (*n* = 10)	T1 vs. T2	T2 vs. T3	T1 vs. T3	T1 (*n* = 27)	T2 (*n* = 26)	T3 (*n* = 25)	T1 vs. T2	T2 vs. T3	T1 vs. T3	T1	T2	T3
Body composition							(*n* = 25)		(*n* = 25)		(*n* = 21)				
Body weight, kg	63.16 ± 10.22	63.22 ± 10.20	62.96 ± 10.33	0.72	0.46	0.59	63.62 ± 9.89	62.47 ± 9.65	62.78 ± 9.48	<0.001	0.11	0.005	0.80	0.84	0.96
Body fat mass, kg	16.08 ± 3.95	16.03 ± 4.71	17.99 ± 8.83	0.63	0.77	0.84	17.50 ± 4.68	16.74 ± 4.39	16.30 ± 4.33	0.002	0.98	0.001	0.56	0.62	0.97
Body fat percentage	25.06 ± 4.81	25.20 ± 5.46	24.42 ± 5.14	1.00	1.00	0.82	27.20 ± 5.46	26.45 ± 5.22	25.52 ± 4.61	0.02	0.53	0.02	0.26	0.44	0.62
Blood parameters							(*n* = 27)	(*n* = 26)	(*n* = 25)						
Liver function related															
AST, U/L	20.80 ± 6.48	21.10 ± 5.28	25.10 ± 12.95	0.92	0.63	0.22	19.37 ± 4.83	20.92 ± 8.49	22.76 ± 9.68	0.45	0.16	0.10	0.78	0.50	0.67
ALT, U/L	24.10 ± 14.31	23.80 ± 10.41	20.40 ± 3.95	0.73	0.31	0.83	23.85 ± 11.88	22.23 ± 13.61	18.76 ± 4.11	0.24	0.69	0.12	0.95	0.38	0.24
AST/ALT	1.04 ± 0.35	1.02 ± 0.34	1.05 ± 0.38	0.67	0.57	0.86	0.94 ± 0.33	1.10 ± 0.38	0.94 ± 0.32	0.003	0.002	0.76	0.35	0.56	0.44
GGT, U/L	50.50 ± 41.20	49.80 ± 41.09	62.60 ± 62.30	0.96	0.28	0.65	34.81 ± 74.16	19.65 ± 21.47	35.24 ± 55.61	0.005	<0.001	0.26	0.02	0.003	0.07
TBIL, µmol/L	13.00 ± 5.14	15.15 ± 3.60	12.53 ± 4.68	0.28	0.22	0.84	12.90 ± 4.45	11.44 ± 2.78	12.83 ± 3.87	0.18	0.07	0.93	0.96	0.004	0.65
Blood sugar related															
GLU, mmol/L	4.04 ± 0.42	4.06 ± 0.59	3.7 ± 0.29	0.92	0.13	0.02	4.10 ± 0.40	4.06 ± 0.89	3.86 ± 0.69	0.62	0.10	0.08	0.76	0.71	0.74
HbA1C, %	5.20 ± 0.42	5.00 ± 0.00	5.20 ± 0.42	0.79	0.35	1.00	5.16 ± 0.43	5.12 ± 0.43	5.08 ± 0.40	0.57	1.00	0.50	0.80	1.00	0.45
Serum lipid															
TRIG, mmol/L	1.58 ± 0.54	1.45 ± 0.78	1.91 ± 1.04	0.49	0.03	0.19	1.21 ± 0.62	1.09 ± 0.52	1.46 ± 0.74	0.06	0.001	0.10	0.08	0.16	0.05
TCHOL, mmol/L	4.26 ± 0.79	4.17 ± 0.85	4.29 ± 0.89	0.68	0.85	0.53	3.83 ± 0.67	3.90 ± 0.55	3.90 ± 0.63	0.47	0.90	0.71	0.09	0.32	0.14
Kidney function related															
CREA, µmol/L	69.70 ± 14.13	69.50 ± 11.84	69.40 ± 11.76	1.00	0.92	1.00	59.37 ± 10.85	69.27 ± 18.87	62.60 ± 10.77	<0.001	0.01	0.01	0.04	0.39	0.10
Urea, µmol/L	4.78 ± 1.19	4.71 ± 1.14	5.00 ± 1.38	0.96	0.47	1.00	4.29 ± 0.88	5.19 ± 1.29	4.80 ± 1.39	<0.001	0.06	0.01	0.14	0.45	0.84
UA, µmol/L	312.3 ± 70.53	325.4 ± 81.15	339.7 ± 101.6	0.43	.44	0.19	273.7 ± 50.97	270.0 ± 52.26	296.7 ± 69.73	0.64	0.01	0.02	0.17	0.10	0.18

1 Each measurement is expressed as mean ± SD. ALT, alanine aminotransferase; AST, aspartate aminotransferase; CREA, creatinine; GGT, γ-glutamyltransferase; GLU, glucose; HbA1C, glycated hemoglobin; T1, before fasting; T2, the end of fasting; T3, 30 d after the end of fasting; TBIL, total bilirubin; TCHOL, total cholesterol; TRIG, triglycerides; UA, uric acid.

2 Wilcoxon signed-rank test was performed for intragroup testing.

3 Mann–Whitney test was performed for intergroup testing.

### Intermittent fasting and microbiome diversity

Taxonomic diversity is an important characteristic of the human microbiome (19), and generally speaking, more diverse microbiomes are considered healthier (20). An often-used measure to quantify diversity in microbiomes is the Shannon–Weaver index (21), and thus we endeavored to determine the effects of intermittent fasting on the ecological diversity of the gut microbiota by quantifying this index in the samples collected. To this end, fecal bacterial compositions were analyzed by 16S ribosomal RNA gene sequencing. Following OTU-calling, it was found that in the young cohort, the diversity of gut microbiota, as indicated by the Shannon–Weaver index and other indices, was statistically significantly increased following intermittent fasting (Figure 1C; **Supplementary Table 1**). In the middle-aged cohort, the Shannon–Weaver index showed a slight upward trend, both compared with controls as well as compared with the pre–intermittent fasting state (Figure 2B), but this effect was not statistically significant (**Supplementary Table 2**), probably because of age-related factors such as immunosenescence and differences in nutrient absorption. These changes in apparent gut microbiome diversity provided a first indication that intermittent fasting affects the composition of the gut microbiome.

### Intermittent fasting remodels the gut microbiome

The notion that the gut microbiome is remodeled during intermittent fasting was confirmed by Bray–Curtis PCA of overall gut microbiota structure after intermitting fasting (the young cohort: ANOSIM test, *P* < 0.001; Figure 1D; the middle-aged cohort: ANOSIM test, *P* < 0.001; Figure 2C). Interestingly, the gut microbial community showed a significant trend (ANOSIM test, *P* < 0.001) of return toward baseline conditions after the discontinuation of fasting (Figure 2C). Further confidence in these data was bolstered by the observation that Bray–Curtis PCA of the non–intermittent fasting control group of the middle-aged cohort showed that microbiome composition did not change in the study period (**Supplementary Figure 5**), also in agreement with the notion that gut microbiomes tend to be stable when lifestyles are not changed (22). We thus concluded that intermittent fasting provoked reversible alterations in the composition of the gut microbiome.

### Intermittent fasting increases butyric acid–producing Lachnospiraceae

**FIGURE 3:**
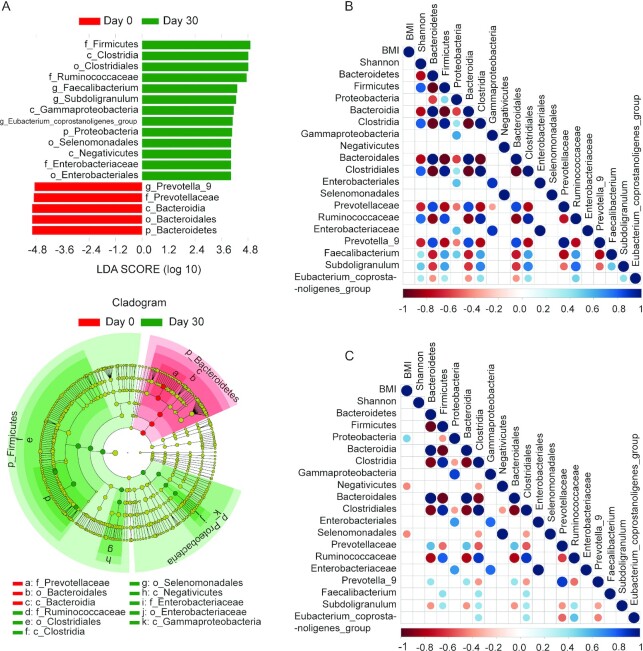
Intermittent fasting–provoked changes of bacterial taxa in the young cohort. (A) Taxa that show alternative abundance before and after fasting are depicted. Taxa with a log linear discriminant analysis (LDA) score above 4.00 as determined by using linear discriminant analysis coupled with effect size measurements (LEfSe). Data shown are the ^10^Log LDA scores following LEfSe analyses; *n* = 30 per time point. The hierarchy of the discriminating taxonomic levels was visualized as cladograms allowing taxonomic comparisons before and after fasting. A correlation matrix between microbiota and BMI before (B) or at the end of fasting (C) is depicted; *n* = 30 per time point. Positive correlations are displayed in blue and negative correlations in red. Color density is proportional to the correlation coefficients (bottom score). The size of the dots is inversely proportional to the *P* value. Only correlations with a *P* value less than 0.05 are shown. All the correlations shown are statistically significant (Spearman correlation, *P* < 0.05).

To further characterize the alternative microbiota taxa abundance associated with intermittent fasting that accounts for the greatest differences before and after fasting, we performed LEfSe analysis (23). As shown in **Figure 3**A, multiple taxonomic differences were found when intermittent fasting in the young adult cohort was contrasted to results from the prefasting microbiome. Among the effects, especially upregulation of OTUs belonging to the phylum Firmicutes was evident, which was driven, among other changes, by an increase in OTUs mapping to the order Clostridiales (**Supplementary Figure 6A; Supplementary Tables 3–4**). Of note, the family Lachnospiraceae did not reach a significance threshold in LEfSe analysis of this cohort. Conversely, OTUs belonging to the phylum Bacteriodes and especially the family Prevotellaceae were reduced following intermittent fasting. These changes appeared more related to the intermittent fasting per se rather than the intermittent fasting–provoked reduction in BMI in general, as no correlation was found between gut microbiota composition and BMI before fasting (Figure 3B). However, we did detect that following fasting, there was a positive correlation between BMI and the abundance of OTUs belonging to the phylum Proteobacteria, whereas after fasting, a negative correlation between BMI and abundance of the class Negativicutes and order Selenomonadales became apparent (Figure 3C).

**FIGURE 4:**
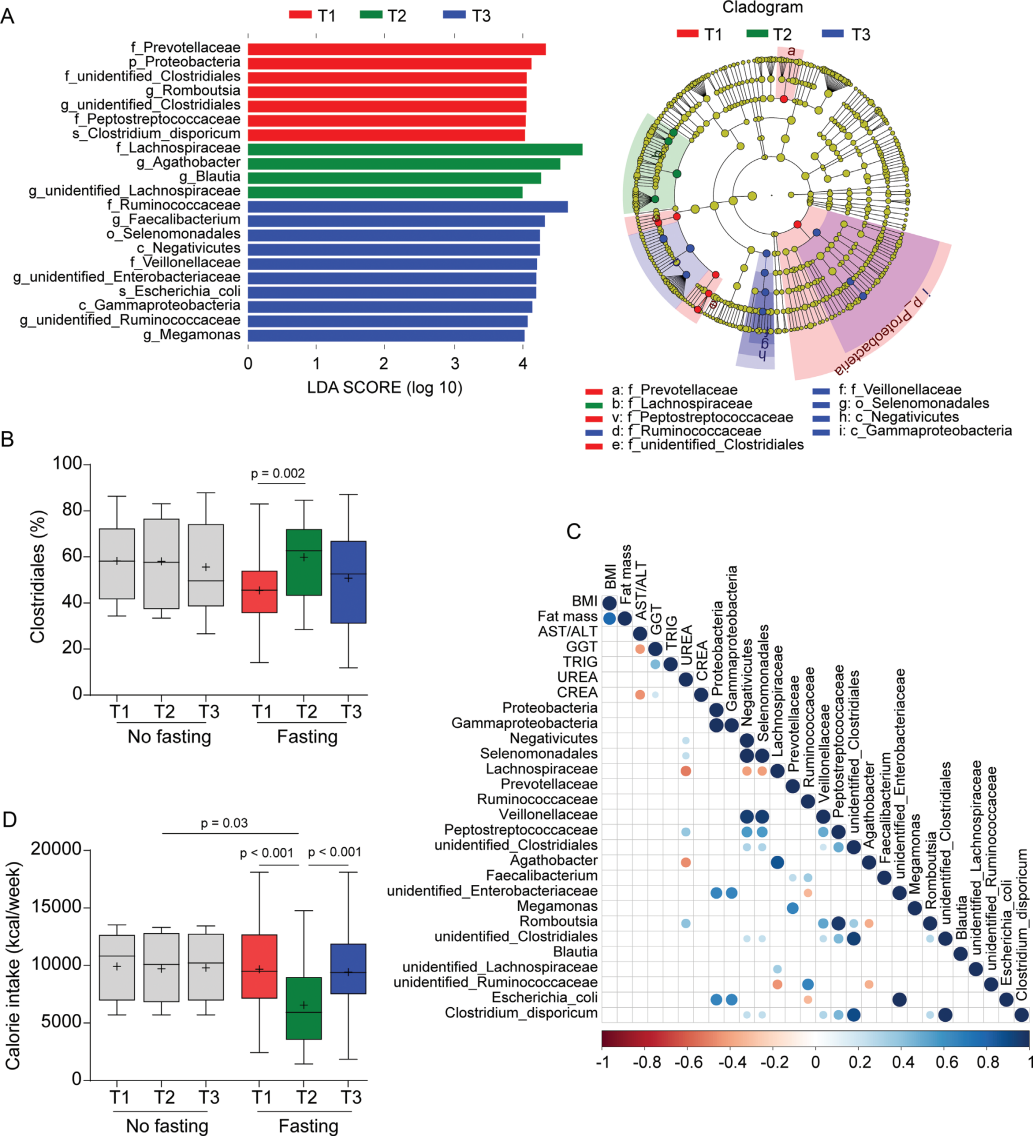
Intermittent fasting–provoked changes of bacterial taxa in the middle-aged cohort. (A) The most differentially abundant taxa at the different time points in fasted participants are shown. Taxa that show alternative abundance at 3 time points were determined using linear discriminant analysis coupled with effect size measurements (LEfSe). The hierarchy of discriminating taxa was visualized by cladograms allowing for taxonomic comparisons between the 3 time points (*n* = 27 for T1 and T2; *n* = 23 for T3). Data shown are the ^10^Log linear discriminant analysis score above 4.00 following LEfSe analyses. (B) Boxplot for relative abundance of the order Clostridiales shows the minimum, the first quantile, median, mean (+), the third quantile, and the maximum abundance values for samples at T1 (*n* = 10 for no fasting compared with *n* = 27 for fasting), T2 (*n* = 10 for no fasting compared with *n* = 27 for fasting), and T3 (*n* = 10 for no fasting compared with *n* = 23 for fasting). Significance between groups was estimated by using a 2-tailed paired Student *t* test intragroup or Mann–Whitney test for intergroup testing. (C) Correlation matrix between microbiota (used in Figure 2E) and changed host physiology (see Table 3) as a consequence of fasting. All the correlations shown are statistically significant (*n* = 26; Spearman correlation, *P* < 0.05). (D) Calorie intake measured by FFQs was reduced during fasting. Boxplot shows the minimum, the first quantile, median, mean (+), the third quantile, and the maximum calorie intake values for samples at 3 time points (no fasting, *n* = 10; fasting, *n* = 23). Significance between groups was estimated by using a 2-tailed paired Student *t* test for intragroup or Mann–Whitney test for intergroup testing.

In the middle-aged cohort, almost no taxa were changed in nonfasting control participants during the study period (**Supplementary Figure 7**). LEfSe analysis of the gut microbiome of the intermittent fasting participants of this middle-aged cohort, however, revealed major shifts in microbiome composition, which were largely similar to those observed in the younger cohort (Figure 4A). Again, the abundance of the order Clostridiales was significantly increased after fasting (Figure 4B), with this effect being dependent on increased abundance of the Lachnospiraceae and Ruminococcaceae families (**Supplementary Tables 5–6**). Concentrations of the former family quickly returned to baseline following cessation of fasting, but increased concentrations of Ruminococcaceae were more resilient in this respect (Supplementary Figure 6B; **Supplementary Figure 8**). Also mirroring the effects seen in the younger cohort, we observed in the intermittent fasting group of the middle-aged cohort decreased abundance of the Prevotellaceae. In this cohort, a correlation between the concentration of serum urea and the abundance of OTUs belonging to the family Lachnospiraceae and genus *Agathobacter* was detectable at the end of fasting (Figure 4C) but not detectable before fasting (**Supplementary Figure 9A**) as well as following cessation of fasting (Supplementary Figure 9B). Functional analysis using PICRUSTs showed 29 pathways that were present and 60 that were absent in the young cohort (**Supplementary Figure 10A**), as well as 14 pathways that were present and 24 that were absent in the middle-aged cohort at the end of fasting when contrasted to pathways present at the start of fasting (Supplementary Figure 10B). Among other observations, the results are in agreement with an increase in mucin degradation-associated metabolic functions in both cohorts during Ramadan. To assess the levels of calorie restriction associated with the effects of intermittent fasting, we estimated the calorie intake via FFQs over the whole course of study. We found that energy intake during the month of fasting was significantly reduced compared with that before or 1 mo after intermittent fasting or with that of unfasted controls (Figure 4D), which fits well with previously published data in this respect (10).

## Discussion

We took advantage of fasting being voluntarily observed by most adherents of the Islamic faith during Ramadan (the ninth month of the Islamic calendar). Ramadan is associated with intermittent fasting for a substantial proportion of humankind (∼20% of the world population), and similar forms of intermittent fasting exist for other important religious faiths as well. Nevertheless, the effects of such altered dietary intake patterns on gut microbiome have not yet been well characterized, but a preliminary study did document shifts in microbiota in Ramadan-adhering participants (24). Importantly, it is recognized that religion-inspired intermittent fasting provokes similar beneficial effects on physiology compared with other forms of intermittent fasting (25). We thus felt that it would be important to characterize the associated changes in gut microbiome.

We observed in 2 independent cohorts, sampled in 2 different years, that Ramadan-associated intermittent fasting induces substantial remodeling of the gut microbiome. Importantly, we established that intermittent fasting in humans is especially associated with an upregulation of butyric acid–producing Lachnospiraceae in a manner that correlates to improvement in human physiologic surrogate markers such as blood glucose and BMI. Intermitting fasting–provoked upregulation of Lachnospiraceae thus may provide a rational explanation for at least some of the beneficial effects reported for intermittent fasting in humans. It should be noted that we failed to include a nonfasting control for the young adult population. This hampers the comparison of the results from the young cohort to an age- and sex-matched nonfasting population.

Although intermittent fasting is regularly practiced for a variety of religious, social, and medical reasons, its effects on physiology remain relatively uncharacterized, and also the potential benefit for human health remains somewhat controversial (23), also in the communication of professional medical organizations to the public at large (e.g., https://www.health.harvard.edu/heart-health/not-so-fast-pros-and-cons-of-the-newest-diet-trend). With respect to the latter, the present study documented beneficial effects in 2 volunteer cohorts, both when prefasting and postfasting states were compared with the fasting period itself, as well as when intermittent fasting participants were compared with closely matched nonfasting controls. In this sense, this study adds further momentum to the body of contemporary biomedical literature supporting intermittent fasting as a healthy intervention. Although alternative microbiome composition has often been considered an important mediator of the effects of intermittent fasting, the data supporting this notion have mainly come from animal experimentation. Our study, however, reports that intermittent fasting alters the composition of gut microbiota in healthy nonobese humans independent of living area and diet composition, but this effect disappeared again when fasting was stopped. Interestingly, some OTUs were still higher in their abundance after the cessation of the fasting, including the family Ruminococcaceae, 3 individual genera (*Faecalibacterium*, unidentified *Enterobacteriaceae*, and unidentified *Ruminococcaceae*), and the species *Escherichia coli*. The reason for this phenomenon is unknown but may be linked to a shorter recovery time after fasting.

These gut microbiota alterations in response to fasting were accompanied by changes in various physiologic parameters and reduced energy intake. Thus, our studies provide strong support for the notion that the human microbiome can be an effector for physiologic effects of intermittent fasting.

Importantly, intermittent fasting causes a reversible upregulation in abundance of members of the Lachnospiraceae family, and this upregulation was correlated to the beneficial physiologic effects of intermittent fasting. The Lachnospiraceae, together with other Clostridiales, are the main source of butyrogenesis in the human intestine (18, 26). Butyric acid is highly bioactive, for instance, stimulating intestinal differentiation (27) through induction of Hedgehog signaling (28), and acts as an important energy source for the epithelial compartment [e.g., Van Den Brink et al. (29)]. Microbiota-generated butyrate is well established to promote metabolic benefits via gut–brain neural circuits (30). In apparent agreement, high concentrations of Lachnospiraceae have been linked to reduced incidence of cancer (31), improvement of inflammatory bowel disease (32), better mental health (33, 34), reduced atopy (35), and better cardiorespiratory fitness (36). It is thus rational to propose that increased abundance of Lachnospiraceae species may mediate the beneficial effects of intermittent fasting on human health, but obviously further work is necessary to substantiate that notion, and until that time, other possibilities should be kept in mind.

The Lachnospiraceae are a family within the order of Clostridiales, apparently ecologically specialized for living in the mammalian gastrointestinal tract (18). Deposited database analysis shows that the family currently contains 24 named genera in addition to various unclassified strains (37) and that all share substantial 16S ribosomal RNA gene sequence similarity, hampering exact classification of the Lachnospiraceae detected in this study. All members of this family are strictly anaerobic bacteria, and maybe because of the specialization of this family to thrive in the mammalian gut, many members of this family appear to have the capacity to ferment mucins (38), which is relatively rare in intestinal ecology (39). In apparent agreement, a PICRUSt analysis showed an increase of mucin degradation-dependent metabolic functions in the microbiome during Ramadan (Supplementary Figure 10). One can envision that the capacity to metabolize mucin provides this family of bacteria a competitive advantage during intermittent fasting when other carbohydrates are not available as an energy source to the flora for prolonged times. In apparent support for this notion was the observation that Lachnospiraceae are not competitive with other flora components in *Muc2*^−/−^ mice, their concentrations being substantially lower compared with Muc2-proficient littermates (40).

In conclusion, the current study has shown that intermittent fasting provokes substantial remodeling of the gut microbiome in healthy nonobese volunteers and that this remodeling involves upregulation of Lachnospiraceae species. As this species is linked to intestinal butyrate production and improved health, this observation provides a rational potential explanation for the beneficial effects linked to intermittent fasting.

## Supplementary Material

nqaa388_Supplemental_File

## Data Availability

Data described in the manuscript will be made publicly and freely available without restriction as supplementary data to this manuscript.

## Notes

Supported by the Gansu Provincial Science and Technology Grant (17YF1WA166, 1504WKCA094), Ministry of Science and Technology Assistance Project Grant (KY201501005), Characteristic Discipline of Bioengineering Construction for the Special Guide Project of the “World-Class Universities and World-Class Disciplines” of Northwest Minzu University (10018703), the Changjiang Scholars and Innovative Research Team in University (IRT_17R88), and the Talent Training Project for Kunming University of Science and Technology (KKSY201560004).

JS and YW share first authorship. ZM and MPP share senior authorship.

This study is registered at http://www.chictr.org.cn/showprojen.aspx?proj=56162 (ChiCTR2000034646).

Supplementary Figures 1–10 and Supplementary Tables 1–6 are available from the “Supplementary data” link in the online posting of the article and from the same link in the online table of contents at https://academic.oup.com/ajcn/.

Abbreviations used: ANOSIM, analysis of similarities; LDA, linear discriminant analysis; LEfSe, linear discriminant analysis coupled with effect size measurements; OTU, operational taxonomic unit; PCA, principal coordinates analysis; PICRUSt, Phylogenetic Investigation of Communities by Reconstruction of Unobserved States; T1, start of the 2018 Ramadan; T2, the end of the 2018 Ramadan; T3, 30 days after the end of the 2018 Ramadan.
