# Supplementary material for: Remodeling of the gut microbiome during Ramadan-associated intermittent fasting
Source: Am J Clin Nutr. 2021 Apr 12;113(5):1332–42. doi: 10.1093/ajcn/nqaa388 (PMC8106760; doi:10.1093/ajcn/nqaa388)
Supplement: nqaa388_Supplemental_File [file nqaa388_supplemental_file.docx]

**On-line Supplementary Material**

**Remodeling of the gut microbiome during Ramadan-associated intermittent fasting**

Junhong Su, Yueying Wang, Xiaofang Zhang, Mingfu Ma, Zhenrong Xie, Qiuwei Pan, Zhongren Ma, Maikel P. Peppelenbosch

**On-line Supplementary Tables**

**Supplementary Table 1** Alpha diversity indices of the gut microbiome in the young cohort before and after intermittent fasting^1^

| Diversity index | Before fasting | After fasting |  |
| --- | --- | --- | --- |
|  | Day 0 (n=30) | Day 30 (n=30) | *P* value^2^ |
| Shannon | 5.17 ± 0.49 | 5.40 ± 0.44 | 0.02 |
| Simpson | 0.92 ± 0.04 | 0.94 ± 0.02 | 0.001 |
| Chao1 | 350.9 ± 34.5 | 372.4 ± 55.5 | 0.04 |
| ACE | 354.4 ± 34.9 | 378.6 ± 56.4 | 0.01 |
| PF whole tree | 48.68 ±11.40 | 37.64 ± 10.20 | <0.001 |
| Goods coverage | 0.99 ± 0.0002 | 0.99 ± 0.0002 | 1 |

^1^Data presented as mean ± SD.

^2^Wilcoxon signed-rank test performed to compare groups.

**Supplementary Table 2** Alpha diversity indices of the gut microbiome in the middle-aged cohort before, after and one month after the cessation of intermittent fasting^1^

| Diversity index | Before fasting | After fasting | Recovery after  fasting |  |  |  |
| --- | --- | --- | --- | --- | --- | --- |
|  | T1 (n=27) | T2 (n=27) | T3 (n=23) |  | *P* value^2^ |  |
|  |  |  |  | T1 vs. T2 | T2 vs. T3 | T1 vs. T3 |
| Shannon | 5.04 ± 0.59 | 5.13 ± 0.59 | 4.95 ± 0.70 | 0.46 | 0.29 | 0.96 |
| Simpson | 0.92 ± 0.06 | 0.92 ± 0.06 | 0.92 ± 0.05 | 0.70 | 1 | 0.38 |
| Chao1 | 352.0 ± 34.3 | 342.9 ± 39.7 | 338.2 ± 55.3 | 0.31 | 0.27 | 0.60 |
| ACE | 350.7 ± 31.1 | 342.8 ± 35.4 | 334.1 ± 49.6 | 0.34 | 0.13 | 0.25 |
| PF whole tree | 21.99 ± 2.10 | 21. 69 ± 2.18 | 22. 13 ± 3.92 | 0.62 | 0.71 | 0.80 |
| Goods coverage | 0.99 ± 0.0005 | 0.99 ± 0.0004 | 0.99 ± 0.0005 | 0.12 | 0.57 | 0.57 |

^1^Data presented as mean ± SD.

^2^Wilcoxon signed-rank test performed to compare groups.

**Supplementary Table 3** OTU number of LEfSe-identified bacterial taxa in the young cohort before and after intermittent fasting^1^

| Taxon | Before fasting | After fasting |  |
| --- | --- | --- | --- |
|  | Day0 (n=30) | Day30 (n=30) | *P* value^2^ |
| Phylum |  |  |  |
| Firmicutes | 25317 ± 8963 | 36305 ± 10300 | <0.001 |
| Bacteroidetes | 33077 ± 10128 | 21994 ± 7902 | <0.001 |
| Proteobacteria | 3496 ± 3029 | 4981 ± 2834 | 0.021 |
| Class |  |  |  |
| Clostridia | 22024 ± 8316 | 31017 ± 10845 | <0.001 |
| Bacteroidia | 33074 ± 10129 | 21993 ± 7902 | <0.001 |
| Negativicutes | 1680 ± 1569 | 3137 ± 2660 | 0.015 |
| Gammaproteobacteria | 1411 ± 2376 | 3202 ± 2430 | <0.001 |
| Order |  |  |  |
| Clostridiales | 22023 ± 8316 | 31016 ± 10845 | <0.001 |
| Bacteroidales | 33074 ± 10129 | 21993 ± 7902 | <0.001 |
| Selenomonadales | 1680 ± 1569 | 3137 ± 2660 | 0.015 |
| Enterobacteriales | 1200 ± 2377 | 2377 ± 1867 | 0.001 |
| Family |  |  |  |
| Ruminococcaceae | 12404 ± 6698 | 19730 ± 8180 | <0.001 |
| Prevotellaceae | 17549 ± 14191 | 8136 ± 10050 | <0.001 |
| Enterobacteriaceae | 1200 ± 2377 | 2377 ± 1867 | 0.001 |
| Genus |  |  |  |
| Prevotella_9 | 13740 ± 11541 | 4815 ± 6344 | <0.001 |
| Faecalibacterium | 3502 ± 1913 | 6134 ± 4141 | 0.001 |
| Subdoligranulum | 1771 ± 1129 | 3985 ± 3100 | <0.001 |
| Eubacterium_coprostanoligenes_group | 949 ± 622 | 2823 ± 3488 | 0.002 |

^1^Data presented as mean ± SD.

^2^Wilcoxon signed-rank test performed to compare groups.

**Supplementary Table 4** Difference in the relative abundance of LEfSe-identified bacterial taxa in the young cohort before and after intermittent fasting^1^

| Taxon | Before fasting | After fasting |  |
| --- | --- | --- | --- |
|  | Day0 (n=30) | Day30 (n=30) | *P* value^2^ |
| Phylum |  |  |  |
| Firmicutes | 40.56± 13.90 | 56.41 ± 14.49 | <0.001 |
| Bacteroidetes | 53.14 ± 15.81 | 34.37 ± 12.51 | <0.001 |
| Proteobacteria | 5.55 ± 4.56 | 7.85 ± 4.53 | 0.035 |
| Class |  |  |  |
| Clostridia | 35.30 ± 12.98 | 48.04 ± 15.17 | <0.001 |
| Bacteroidia | 53.14 ± 15.82 | 34. 36± 12.51 | <0.001 |
| Negativicutes | 2.67 ± 2.45 | 4.95 ± 4.20 | 0.017 |
| Gammaproteobacteria | 2.26 ± 3.64 | 5.08 ±3.93 | <0.001 |
| Order |  |  |  |
| Clostridiales | 35.30 ± 12.98 | 48.04 ± 15.17 | <0.001 |
| Bacteroidales | 53.14 ± 15.82 | 34.36 ± 12.51 | <0.001 |
| Selenomonadales | 2.67 ± 2.45 | 4.95 ± 4.20 | 0.017 |
| Enterobacteriales | 1.91 ± 3.62 | 3.78 ± 3.12 | <0.001 |
| Family |  |  |  |
| Ruminococcaceae | 19.88 ± 10.56 | 30.43 ± 11.41 | <0.001 |
| Prevotellaceae | 27.81 ± 21.60 | 12.92 ± 16.11 | <0.001 |
| Enterobacteriaceae | 1.91 ± 3.62 | 3.78 ± 3.12 | <0.001 |
| Genus |  |  |  |
| Prevotella_9 | 21.79 ± 17.69 | 7.63 ± 10.20 | <0.001 |
| Faecalibacterium | 5.62 ± 2.96 | 9.47 ± 5.93 | 0.001 |
| Subdoligranulum | 2.85 ± 1.82 | 6.15 ± 4.57 | <0.001 |
| Eubacterium_coprostanoligenes_group | 1.51 ± 0.99 | 4.27 ± 5.20 | 0.002 |

^1^Data presented as mean ± SD.

^2^Wilcoxon signed-rank test performed to compare groups.

**Supplementary Table 5** OTU number of LEfSe-identified bacterial taxa in the middle-aged cohort before, after and one month after the cessation of intermittent fasting^1^

| Taxon | Before fasting | After fasting | Recovery after  fasting |  |  | |  | |
| --- | --- | --- | --- | --- | --- | --- | --- | --- |
|  | T1 (n = 27) | T2 (n = 27) | T3 (n = 23) |  | *P* value^2^ |  | |  |
|  |  |  |  | T1 vs. T2 | T2 vs. T3 | T1 vs. T3 | |  |
| Phylum |  |  |  |  |  |  | |  |
| Proteobacteria | 5599 ± 6817 | 3342 ± 5166 | 5258 ± 6293 | 0.12 | 0.13 | 0.80 | |  |
| Class |  |  |  |  |  |  | |  |
| Gammaproteobacteria | 4983 ± 6153 | 3213 ± 5124 | 5184 ± 6305 | 0.15 | 0.11 | 0.60 | |  |
| Negativicutes | 3023 ± 3614 | 1043 ± 1876 | 4067 ± 6316 | 0.006 | 0.003 | 0.62 | |  |
| Order |  |  |  |  |  |  | |  |
| Selenomonadales | 3023 ± 3614 | 1043 ± 1876 | 4067 ± 6316 | 0.006 | 0.003 | 0.62 | |  |
| Family |  |  |  |  |  |  | |  |
| Lachnospiraceae | 16754 ± 9369 | 27691 ± 11782 | 15538 ± 11311 | 0.001 | <0.001 | 0.31 | |  |
| Peptostreptococcaceae | 2836 ± 5235 | 1656 ± 1754 | 1194 ± 1765 | 0.97 | 0.17 | 0.13 | |  |
| Prevotellaceae | 6483 ± 7577 | 5313 ± 10616 | 6436 ± 9218 | 0.06 | 0.58 | 0.17 | |  |
| Ruminococcaceae | 9109 ± 4620 | 11296 ± 6491 | 15183 ± 8394 | 0.08 | 0.008 | <0.001 | |  |
| unidentified_Clostridiales | 1584 ± 3507 | 297 ± 273 | 1290 ± 4262 | <0.001 | 0.65 | 0.07 | |  |
| Veillonellaceae | 2689 ± 3677 | 825 ± 1806 | 3630 ± 6393 | 0.003 | 0.007 | 0.50 | |  |
| Genus |  |  |  |  |  |  | |  |
| Agathobacter | 3639 ± 2970 | 8847 ± 9789 | 3607 ± 5330 | 0.01 | <0.001 | 0.25 | |  |
| Blautia | 4277 ± 4483 | 6719 ± 3812 | 3592 ± 4156 | 0.01 | 0.002 | 0.80 | |  |
| Faecalibacterium | 4811 ± 3650 | 5341 ± 3013 | 7866 ± 5228 | 0.51 | 0.004 | 0.002 | |  |
| Megamonas | 1029 ± 2438 | 25 ± 66 | 1817 ± 5837 | <0.001 | <0.001 | 0.51 | |  |
| Romboutsia | 2459 ± 5204 | 1262 ± 1499 | 781 ± 1606 | 0.75 | 0.03 | 0.05 | |  |
| unidentified_Clostridiales | 1464 ± 3511 | 219 ± 253 | 321 ± 498 | <0.001 | 0.87 | 0.02 | |  |
| unidentified_Enterobacteriaceae | 2498 ± 5059 | 1825 ± 3064 | 4330 ± 5780 | 0.71 | 0.09 | 0.03 | |  |
| unidentified_Lachnospiraceae | 2919 ± 2333 | 4400 ± 1844 | 2570 ± 1445 | 0.01 | <0.001 | 0.54 | |  |
| unidentified_Ruminococcaceae | 1411 ± 1081 | 1744 ± 1259 | 2898 ± 2412 | 0.14 | 0.03 | <0.001 | |  |
| Species |  |  |  |  |  |  | |  |
| *Clostridium_disporicum* | 1356 ± 3524 | 177 ± 232 | 268 ± 468 | <0.001 | 0.69 | 0.04 | |  |
| *Escherichia_coli* | 2497 ± 5059 | 1825 ± 3064 | 4325 ± 5764 | 0.71 | 0.09 | 0.03 | |  |

^1^Data presented as mean ± SD.

^2^Wilcoxon signed-rank test performed to compare groups.

**Supplementary Table 6** Difference in the relative abundance of LEfSe-identified bacterial taxa in the middle-aged cohort before, after and one month after the cessation of intermittent fasting^1^

| Taxon | Before fasting | After fasting | Recovery after  fasting |  |  |  |
| --- | --- | --- | --- | --- | --- | --- |
|  | T1 (n = 27) | T2 (n = 27) | T3 (n = 23) |  | *P* value^2^ |  |
|  |  |  |  | T1 vs. T2 | T2 vs. T3 | T1 vs. T3 |
| Phylum |  |  |  |  |  |  |
| Proteobacteria | 8.20 ± 9.92 | 4. 82 ±7.49 | 7.98 ± 9.92 | 0.12 | 0.086 | 0.87 |
| Class |  |  |  |  |  |  |
| Gammaproteobacteria | 7.26 ± 8.77 | 4.63 ± 7.49 | 7.87 ± 9.93 | 0.13 | 0.08 | 0.64 |
| Negativicutes | 4.53 ± 5.46 | 1.52 ± 2.84 | 6.09 ±9.20 | 0.003 | <0.001 | 0.63 |
| Order |  |  |  |  |  |  |
| Selenomonadales | 4.53 ±5.46 | 1.52 ± 2.84 | 6.07 ± 9.20 | 0.003 | <0.001 | 0.64 |
| Family |  |  |  |  |  |  |
| Lachnospiraceae | 24.6± 13.67 | 39.66 ±15.92 | 23.37 ± 14.94 | 0.002 | <0.001 | 0.56 |
| Peptostreptococcaceae | 4.12 ± 7.34 | 2.40 ± 2.60 | 1.75 ± 2.38 | 0.59 | 0.20 | 0.13 |
| Prevotellaceae | 9.61 ± 11.34 | 7.54 ± 14.86 | 9.15 ± 12.60 | 0.070 | 0.52 | 0.07 |
| Ruminococcaceae | 13.39 ± 6.89 | 16.30 ± 9.09 | 23.17 ± 12.90 | 0.15 | 0.005 | <0.001 |
| unidentified_Clostridiales | 2.48 ±5.89 | 0.43 ± 0.38 | 1.90 ± 6.09 | <0.001 | 0.62 | 0.12 |
| Veillonellaceae | 4.03 ± 5.55 | 1.20 ± 2.75 | 5.40 ± 9.31 | 0.003 | 0.004 | 0.54 |
| Genus |  |  |  |  |  |  |
| Agathobacter | 5.42 ± 4.56 | 12.57 ± 13.75 | 5.34 ± 7.05 | 0.02 | 0.002 | 0.41 |
| Blautia | 6.19 ± 6.36 | 9.71 ± 5.66 | 5.37 ± 5.46 | 0.01 | 0.003 | 0.91 |
| Faecalibacterium | 7.08 ± 5.59 | 7.82 ± 4.74 | 11.82 ± 7.69 | 0.66 | 0.009 | 0.002 |
| Megamonas | 1.53 ± 3.45 | 0.04 ± 0.86 | 2.58 ± 8.21 | 0.01 | 0.003 | 0.92 |
| Romboutsia | 3.57 ± 7.30 | 1.84 ± 2.23 | 1.09 ± 2.03 | 0.61 | 0.06 | 0.04 |
| unidentified_Clostridiales | 2.31 ± 5.90 | 0.31 ± 0.35 | 0.51 ± 0.88 | <0.001 | 0.63 | 0.03 |
| unidentified_Enterobacteriaceae | 3.64 ± 7.18 | 2.60 ± 4.29 | 6.55 ± 9.03 | 0.73 | 0.05 | 0.028 |
| unidentified_Lachnospiraceae | 4.32 ± 3.48 | 6.30 ± 2.50 | 4.10 ± 2.52 | 0.01 | 0.002 | 0.62 |
| unidentified_Ruminococcaceae | 2.06 ± 1.51 | 2.53 ± 1.84 | 4.55 ± 4.05 | 0.16 | 0.02 | <0.001 |
| Species |  |  |  |  |  |  |
| *Clostridium_disporicum* | 2.16 ± 5.92 | 0.25 ± 0.32 | 0.43 ± 0.84 | <0.001 | 0.46 | 0.05 |
| *Escherichia_coli* | 3.64 ± 7.18 | 2.60 ± 4.29 | 6.54 ± 9.00 | 0.73 | 0.05 | 0.03 |

^1^Data presented as mean ± SD.

^2^Wilcoxon signed-rank test performed to compare groups.

**On-line Supplementary Figures**

**
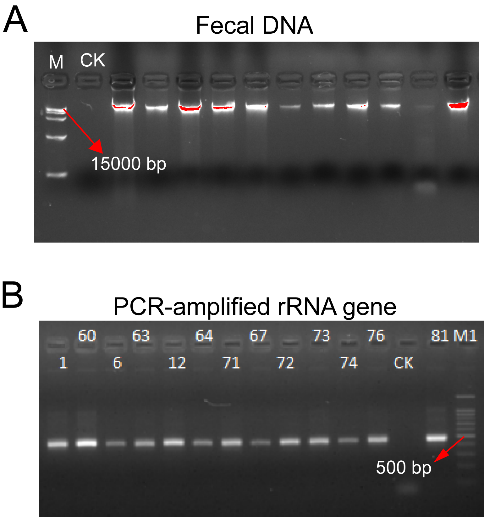
**

**Supplementary Figure 1.** DNA quality check by agarose gel. Fecal DNA (A) and PCR-amplified rRNA gene (B) was checked on 1% agarose gel. A blank control (CK) was taken during DNA isolation or PCR amplification in order to characterize laboratory contamination and kitome. The result showing PCR product (figure 1B) was provided by the Novogene Company (Beijing, China). M: 15000pb DNA ladder. M1, 100 bp DNA ladder. In the present study, the influence of laboratory contamination and kitome was not considered/neglected if no DNA bands or target specific bands were visible in blank controls for fecal DNA isolation or PCR amplification.

**
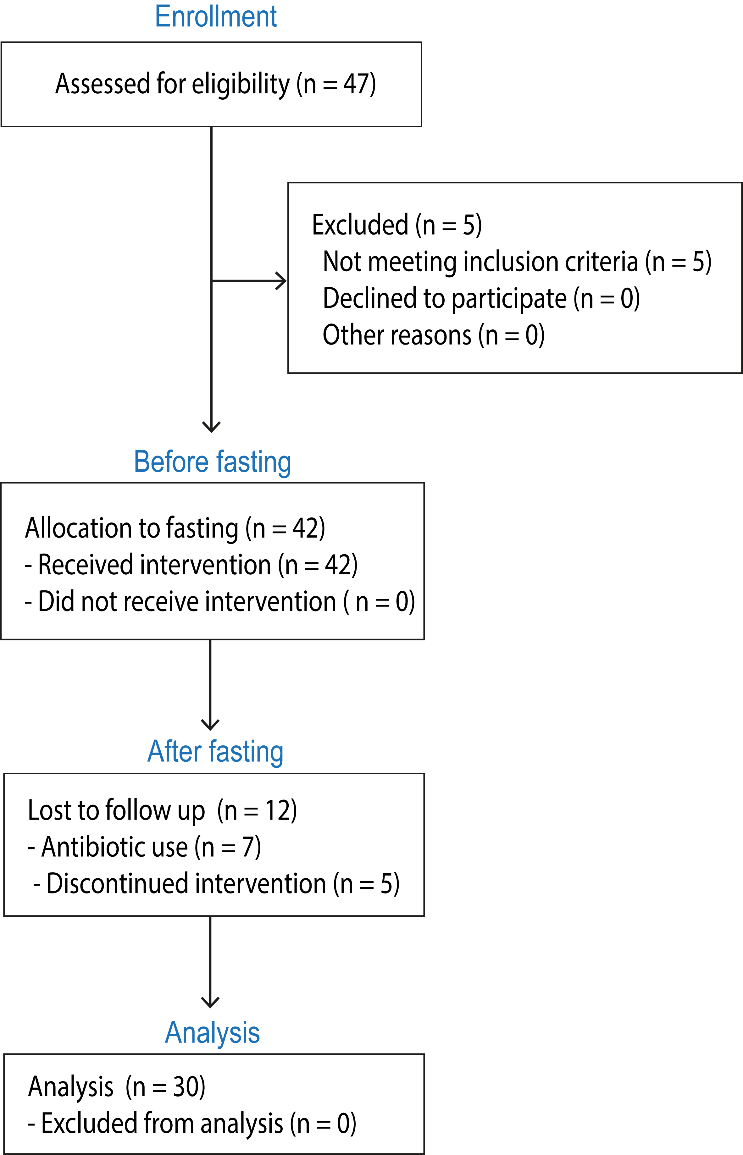
**

**Supplementary Figure 2**. Consort flow diagram indicating the study design and number of participants at the young cohort. After interviewing 47 young adults for study eligibility, 42 study participants without a self-reported medical history in chronic diseases were eventually selected. Other inclusion criteria were a body mass index (BMI) between 18.5 and 29.9 kg/m², and absence of antibiotic use in the month prior to the study. During these 30 days of Ramadan, 12 participants dropped out due to antibiotic use (n = 7) or unwillingness to continue (n = 5). In this young cohort, body weight and height, age and sex were recorded.


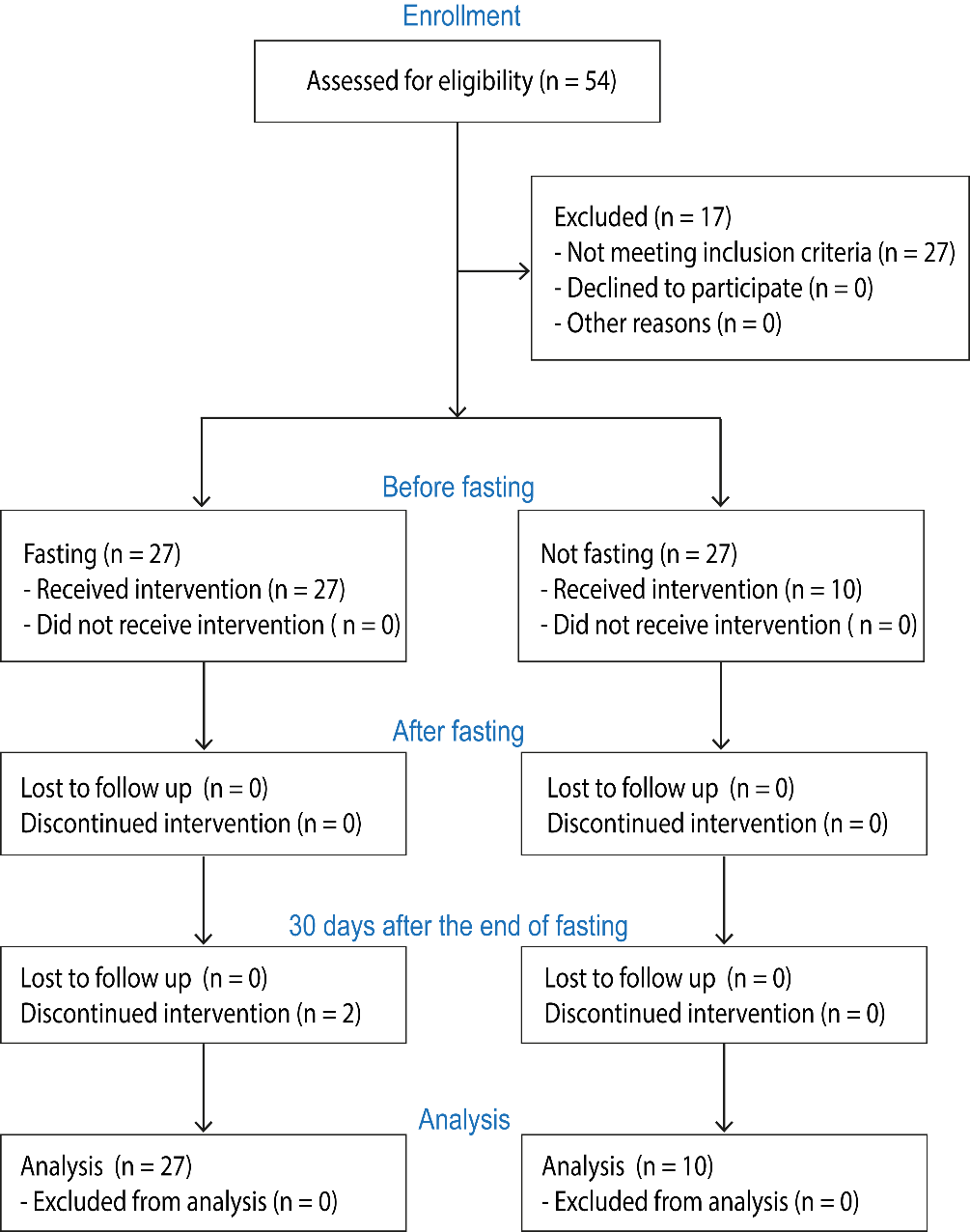


**Supplementary Figure 3.** Consort flow diagram indicating the study design and number of participants at validated middle-aged cohort. After interviewing 54 adults for study eligibility based on the evaluation of their medical history, thirty-seven healthy non-obese participants not using antibiotics in the month up to the start of the study were finally enrolled in this cohort. Twenty-seven of them who intended to observe fasting during Ramadan were included to the intermittent fasting group, ten participants who followed standard diet served as no fasting controls. During these 30 days of Ramadan, no participants dropped out. During follow-up in the post Ramadan period, two participants refused to continue with the study because of unknown reasons. In this middle-aged cohort, body composition (weight, height, body fat mass and body fat percentage), age, sex, and blood parameters (aspartate aminotransferase, alanine aminotransferase, the ratio of aspartate aminotransferase to alanine aminotransferase, gamma glutamyl transferase, total bilirubin, fasting blood glucose, Hemoglobin A1c, triglycerides, total cholesterol, creatinine, urea and uric acid) were recorded and measured. Information on food intake was collected as well.

**
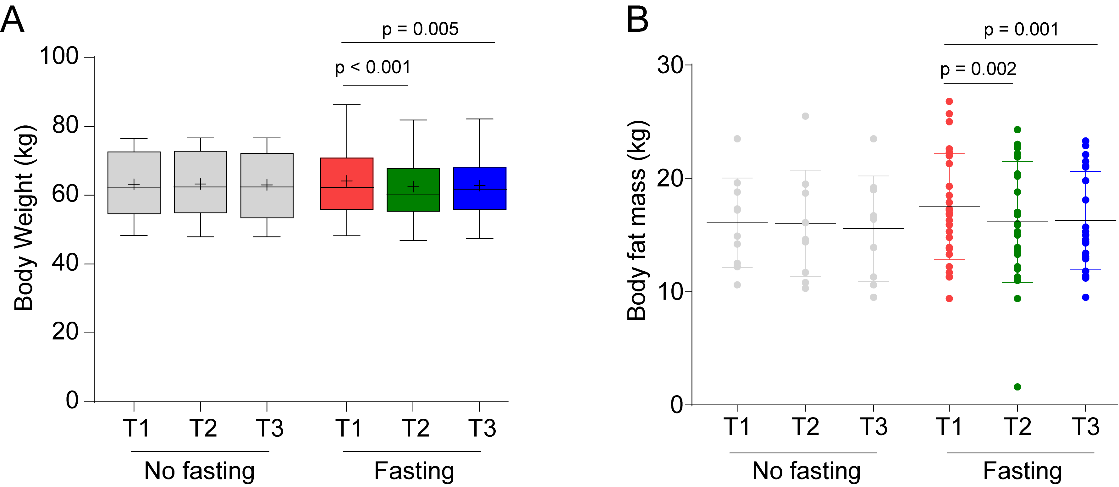
**

**Supplementary Figure 4.** Changes in body weight and body fat during Ramadan-associated intermittent fasting. (A) Box plot for body weight shows the minimum, the first quantile, median, mean (+), the third quantile and the maximum weight values for samples at T1 (before Ramadan; n = 10 for no fasting vs. n = 27 for fasting), T2 (after Ramadan; n = 10 for no fasting vs. n = 27 for fasting) and T3 (30 days after the end of Ramadan; n = 10 for no fasting vs. n = 25 for fasting). (B) Body fat mass at T1 (n = 10 for no fasting vs. n = 25 for fasting), T2 (n = 10 for no fasting vs. n = 25 for fasting) and T3 (n = 9 for no fasting vs. n = 21 for fasting). Data are mean ± SD. Significance between groups was estimated by using a two-tailed paired Student’s *t* test for intragroup testing or Mann Whitney test for intergroup testing.


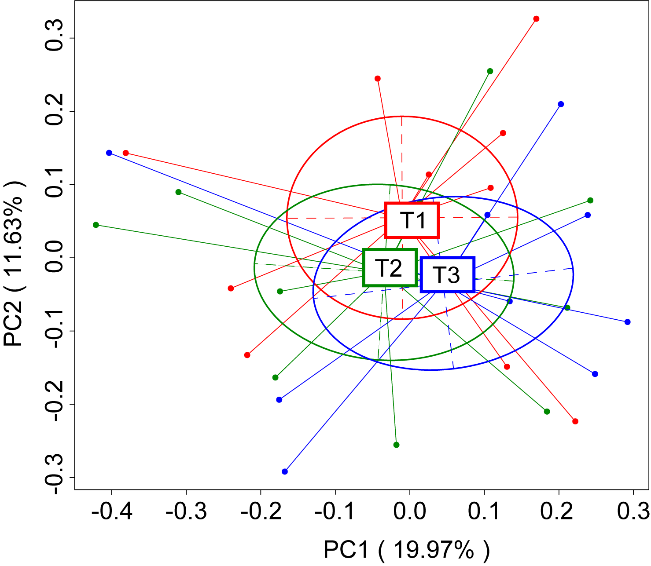


**Supplementary Figure 5.** Principal Co-ordinates Analysis (PCoA) on Bray-Curtis dissimilarities of bacterial communities from unfasted participants at three time points. Each point corresponds to a community from a single individual. Colors indicate community identity. Ellipses show the 95% confidence intervals. Difference in community shift was indicated by using ANOSIM test (n = 10 per time point). No difference was found between T1 (before Ramadan), T2 (after Ramadan) and T3 (30 days after the end of Ramadan).


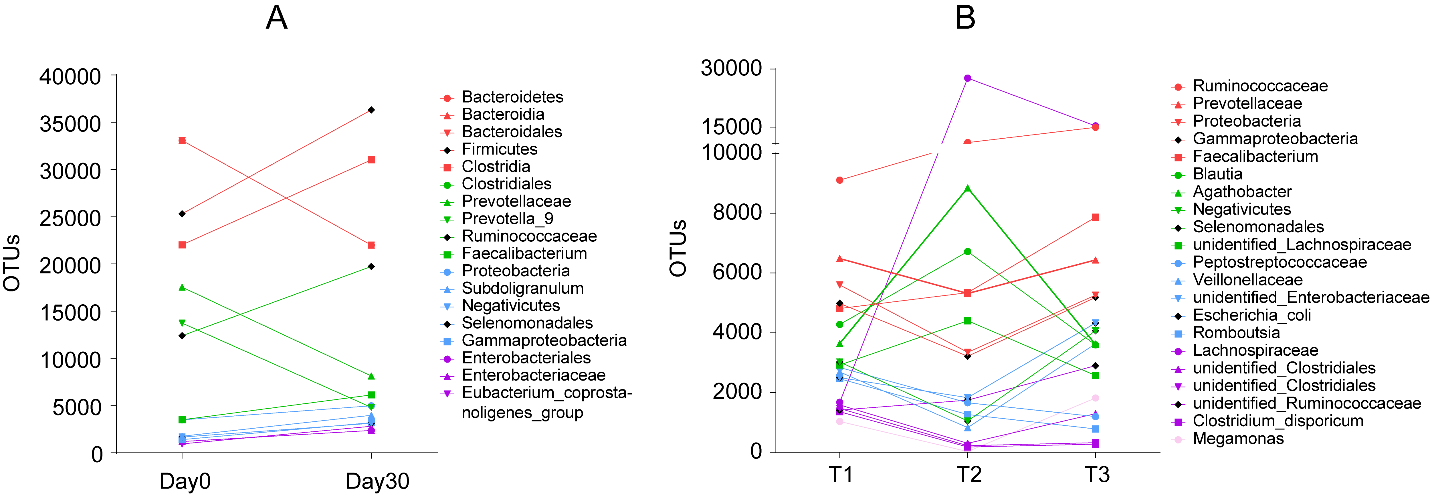


**Supplementary Figure 6.** Mean OTU number of each taxon that is identified by LEfSe (A) in the young cohort on day 0 (before Ramadan; n = 30) and day 30 (after Ramadan; n = 30) and (B) in the middle-aged cohort at T1 (before Ramadan; n = 27), T2 (after Ramadan; n = 27) and T3 (30 days after the end of Ramadan; n = 23). Statistical analysis is shown in Supplementary Table 3 and 5, respectively.
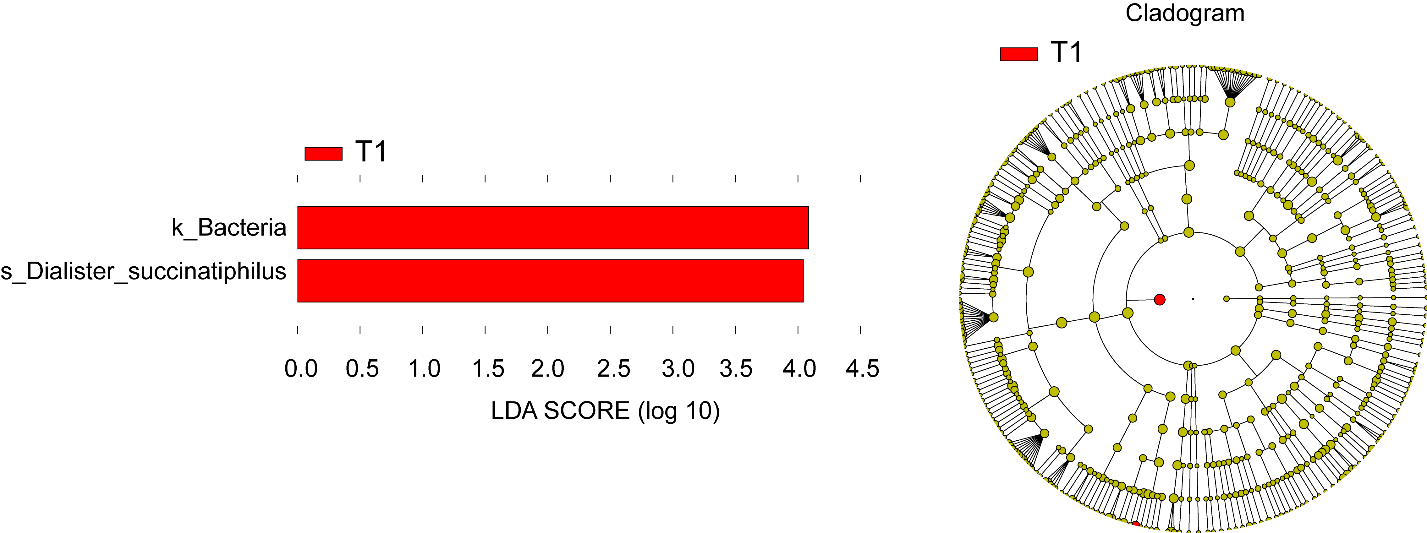


**Supplementary Figure 7.** The most differentially abundant taxa in unfasted participants (n = 10 per time point). Taxa that discriminated at T1 (before Ramadan), T2 (after Ramadan) and T3 (30 days after the end of Ramadan) were determined by using LEfSe. Taxa with a log LDA (linear discriminant analysis) score above 4.00 as determined by using LEfSe. Data shown are the ^10^log LDA scores following LEfSe analyses and the hierarch of discriminating taxa visualized as cladograms for taxonomic comparisons between three time points. There were no differentially abundant taxa in unfasted participants either after or one month after the cessation of fasting.


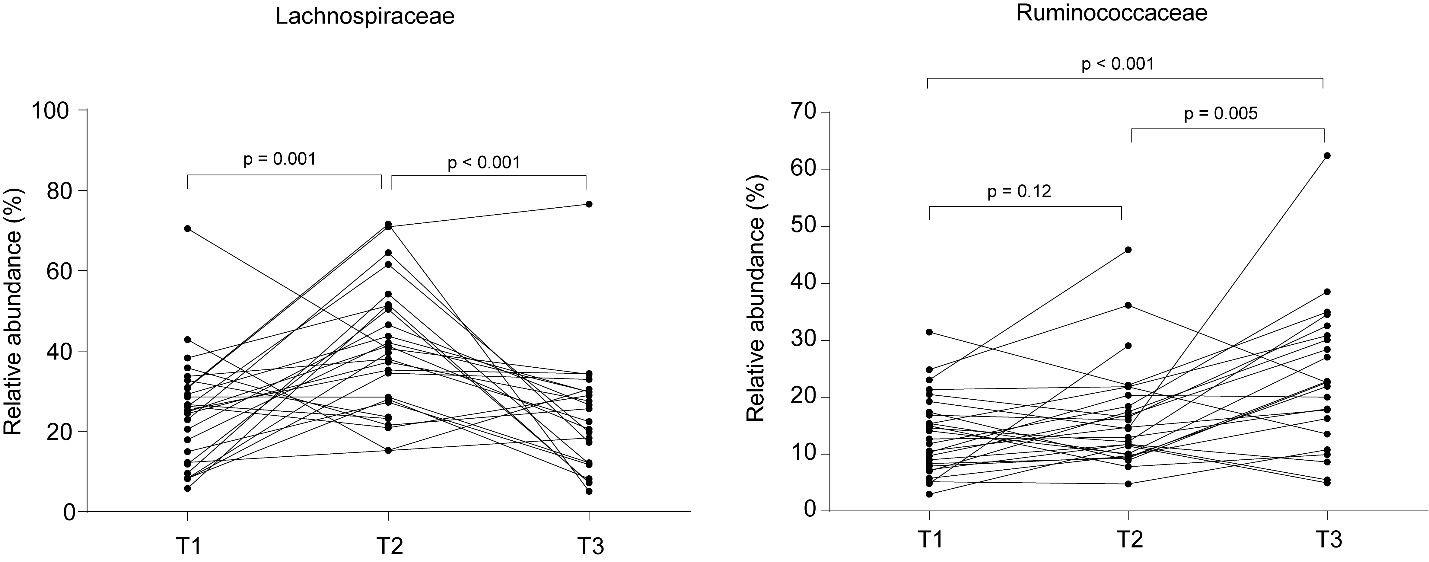
**Supplementary Figure 8**. Relative abundance of the families Lachnospiraceae and Ruminococcaceae for each individual in the middle-aged cohort at T1 (before Ramadan; n = 27), T2 (after Ramadan; n = 27) and T3 (30 days after the end of Ramadan; n = 23). Significance between time points was estimated by using a two-tailed paired student’s *t* test.


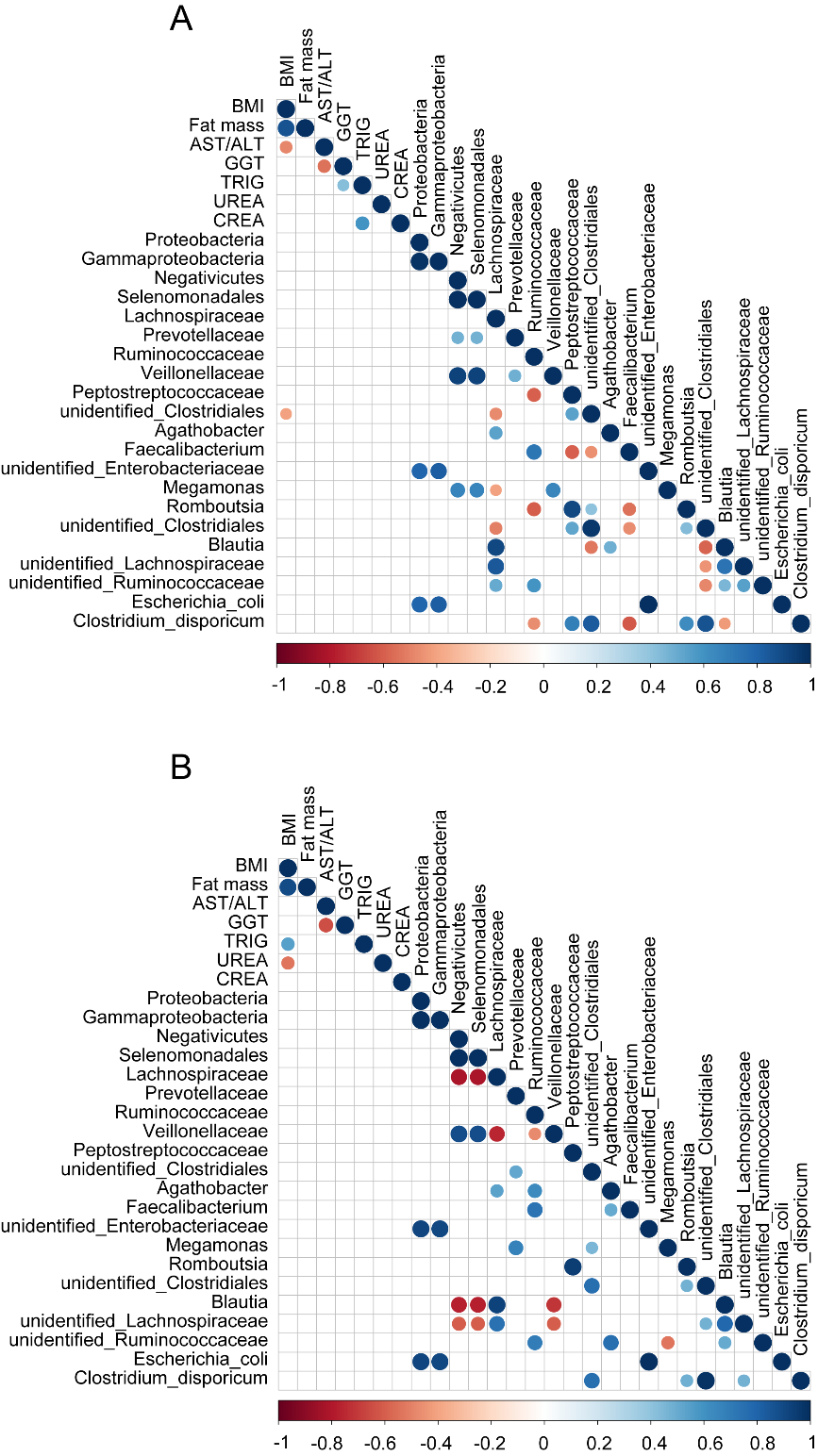


**Supplementary Figure 9.** Correlation matrix between microbiota (used in Figure 2E) and host markers (used in TABLE 3). (**A**) Correlation analysis before Ramadan-associated intermittent fasting (n = 27). (**B**) Correlation analysis of microbiota determined 30 days following the end of Ramadan (n = 23). Positive correlations are displayed in blue and negative correlations in red. Color density is proportional to the correlation coefficients (bottom scare). The size of the dots is inversely proportional of the P-value. Only correlations with p-value less than 0.05 are shown. All the correlations shown are statistically significant (Spearman correlation, p < 0.05).


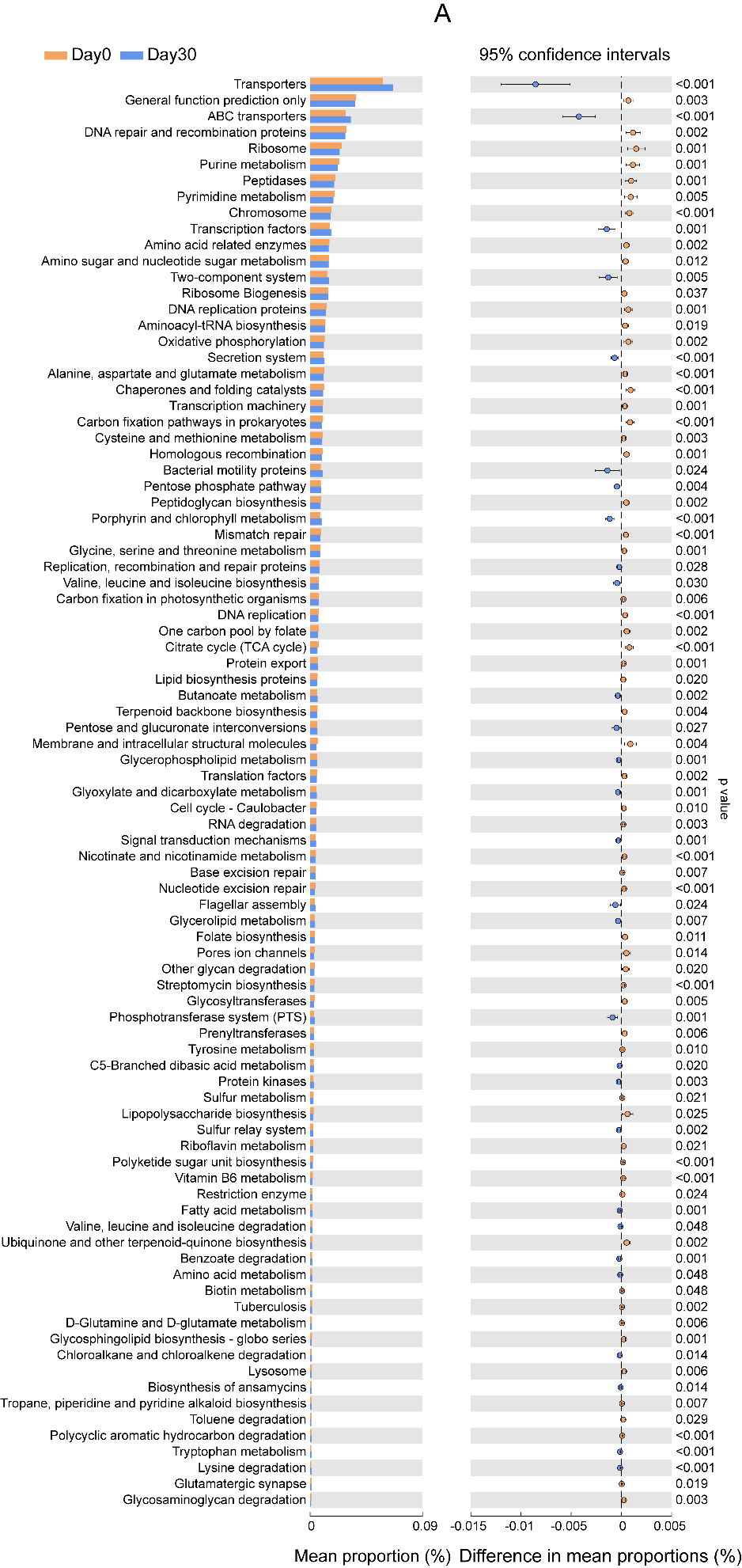


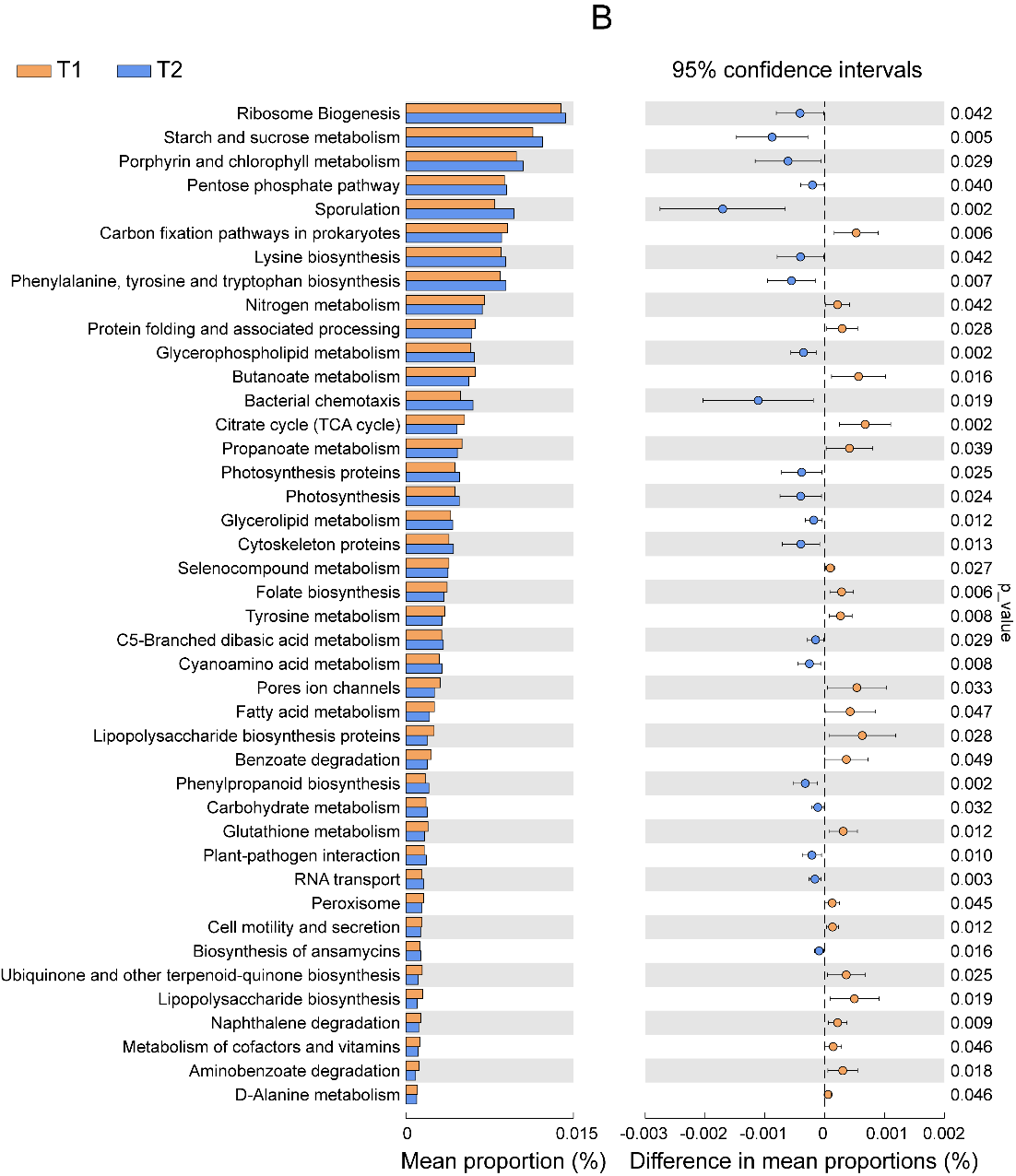


**Supplementary Figure 10**. PICRUSt analysis of KEGG pathways. Predicted metagenomics pathways based on 16S rRNA gene sequencing data from (A) the young cohort on day 0 (before Ramadan; n = 30) and day 30 (after Ramadan; n = 30), and from (B) the middle-aged cohort at T1 (before Ramadan; n = 27), T2 (after Ramadan; n = 27) and T3 (30 days after the end of Ramadan; n = 23). Data are shown as mean proportions (%). Significant KEGG pathways at level 3 were identified by two-side *t* test and shown when a p*-*value is above 0.05 with 95% CIs.
